# Genome Size Versus Genome Assemblies: Are the Genomes Truly Expanded in Polyploid Fungal Symbionts?

**DOI:** 10.1093/gbe/evaa217

**Published:** 2020-10-14

**Authors:** Pepijn W Kooij, Jaume Pellicer

**Affiliations:** e1 Department of Comparative Plant and Fungal Biology, Royal Botanic Gardens, Kew, Richmond, United Kingdom; e2 Center for the Study of Social Insects, São Paulo State University (UNESP), Rio Claro, Sao Paulo, Brazil; e3 Institut Botànic de Barcelona (IBB, CSIC-Ajuntament de Barcelona), Barcelona, Spain

**Keywords:** evolution, fungi, fungus-growing ants, genome assembly, genome size, mutualism

## Abstract

Each day, as the amount of genomic data and bioinformatics resources grows, researchers are increasingly challenged with selecting the most appropriate approach to analyze their data. In addition, the opportunity to undertake comparative genomic analyses is growing rapidly. This is especially true for fungi due to their small genome sizes (i.e., mean 1C = 44.2 Mb). Given these opportunities and aiming to gain novel insights into the evolution of mutualisms, we focus on comparing the quality of whole genome assemblies for fungus-growing ants cultivars (Hymenoptera: Formicidae: Attini) and a free-living relative. Our analyses reveal that currently available methodologies and pipelines for analyzing whole-genome sequence data need refining. By using different genome assemblers, we show that the genome assembly size depends on what software is used. This, in turn, impacts gene number predictions, with higher gene numbers correlating positively with genome assembly size. Furthermore, the majority of fungal genome size data currently available are based on estimates derived from whole-genome assemblies generated from short-read genome data, rather than from the more accurate technique of flow cytometry. Here, we estimated the haploid genome sizes of three ant fungal symbionts by flow cytometry using the fungus *Pleurotus ostreatus* (Jacq.) P. Kumm. (1871) as a calibration standard. We found that published genome sizes based on genome assemblies are 2.5- to 3-fold larger than our estimates based on flow cytometry. We, therefore, recommend that flow cytometry is used to precalibrate genome assembly pipelines, to avoid incorrect estimates of genome sizes and ensure robust assemblies.

Significance

Genome sequencing and analyses are increasing daily due to decreasing costs; however, analyzing the data can be difficult at times due to a large availability of software potentially leading to erroneous genome assemblies. In our study, we show that different software can lead to different conclusion for the same genome data, that is, when the genome assembly is longer the number of genes one can predict from that assembly increases as well. We show that by accurately measuring the genome size using flow cytometry, the resulting data can help as a quality control for the genome assemblies.

## Results and Discussion 

Mutualistic symbioses, in which both partners benefit from living with each other, are an important driver for the evolution of biodiversity (Thompson 2005). To further understand the mechanisms underpinning this biodiversity as well as the evolution of mutualisms themselves, it is important to compare mutualistic species with their nonmutualistic relatives. Thanks to the decreasing costs of whole-genome sequencing (Wetterstrand 2019) it has become increasingly easy to tackle these kinds of comparisons at the genomic level (Nygaard et al. 2016; Knapp et al. 2018). For example, in a recent study using comparative genomics it was shown that the genomes of ericoid mycorrhizal fungi, that form a mutualistic relationship with plants from the family Ericaceae, are more similar to saprotrophic fungi than other mutualistic ectomycorrhizal fungi (Martino et al. 2018). In another study, comparing rates of sequence divergence in the genomes of ants that form mutualistic relationships with plants with those of their nonmutualistic relatives, it was shown that rates of molecular evolution were higher in mutualistic ants, suggesting similar selective pressures to those of parasites (Rubin and Moreau 2016).

Although the decreasing sequencing costs have enabled more researchers to generate genomic data (Stephens et al. 2015), it has also led to the development of an increasing number of bioinformatic tools for analyzing the data (Clément et al. 2018). Researchers are thus faced with the challenge of selecting the most appropriate bioinformatic pipelines to ensure accurate genome assemblies with sufficient sequencing depth to accurately capture the full genomic diversity (Sims et al. 2014). As a result, it has become a relatively common practice to use the default parameters (e.g., k-mer size, PHRED quality offset, bubble algorithms, and overlap sizes) in these bioinformatic resources (Yang et al. 2011; Lai et al. 2014; Larriba et al. 2014; Liu et al. 2014; Wang et al. 2014; Quandt et al. 2015; Dentinger et al. 2016; de Man et al. 2016; Nygaard et al. 2016; Teixeira et al. 2017; Sun et al. 2018; Pucker 2019), rather than checking whether the fine-tuning of these parameters is necessary to increase the accuracy of the genome assembly.

Research into the role of the fungal symbiont of fungus-growing ants has a long history and has become a model system for studying mutualisms (Wheeler 1907; Weber 1979; Mueller et al. 2005). Briefly, the fungus provides the ants with a stable food source whereas the ants in return provide the fungus with active dispersal vectors in the form of flying queens, protection and grooming, and suitable growth substrate (Mueller et al. 2005). Generally, this type of mutualism can be divided into several “agricultural” systems; 1) Basal agriculture, the oldest group with small ant-fungal colonies of ∼100 workers, and comprising fungal species/cultivars mostly from the genus *Leucocoprinus* Pat. (1888) that are typically dikaryotic (i.e., each cell is functionally diploid as it contains two different haploid nuclei); 2) Domesticated agriculture, with colony sizes typically ranging from a few hundred to a few thousand workers and containing heterokaryotic fungi (i.e., each cell is functionally autopolyploid, since although it contains multiple haploid nuclei, there are still just two distinct genomes), mostly from the genus *Leucoagaricus* Locq. ex Singer (1948); 3) Leaf-cutting agriculture, with colony sizes ranging from thousands to several millions of workers associated with just one type of fungus (*Leucoagaricus gongylophorus* [Möller] Singer (1986)) with cells that are multinucleate and multigenomic (i.e., functionally allopolyploid, with each cell containing multiple nuclei with, on average, seven distinct genomes) (Mehdiabadi and Schultz 2010; Kooij et al. 2015). Because these fungi are genetically highly heterozygous and, in some cases, as described above, functionally polyploid, it has been difficult to assemble their genomes, the assemblies are, therefore, often highly fragmented (Aylward et al. 2013). Furthermore, published genome assembly sizes for these fungi are much larger (i.e., >100 Mb) compared with the average fungal genome size of 44.2 Mb for all fungi (1,850 species analyzed, Ramos et al. 2015) and ∼50 Mb for Agaricales (based on data for 11 species, Gupta et al. 2018), the order to which these fungi belong. In order to see whether the existing fragmented genome assemblies have overestimated the genome sizes of these fungi, and to help optimize the parameters used by genome assemblers to increase genome contiguity, we generated robust genome size estimates with flow cytometry and compared them with genome sizes estimated from different genome assemblers.

We sampled material from the Fungarium at the Royal Botanic Gardens, Kew, for *Leucoagaricus barssii* (Zeller) Vellinga (2000) (KM164561), a free-living relative of the fungus-growing ant fungi and the type species for the genus *Leucoagaricus*. The fungal symbiont of the ant *Cyphomyrmex costatus* Mann 1922 (MS140512-07) was isolated and grown on Potato Dextrose Agar (Sigma-Aldrich, St Louis, MO) with added Yeast Extract (Thermo Fisher Scientific Oxoid Ltd, Basingstoke, UK). DNA was extracted from both samples using the QIAgen DNeasy Plant Tissue kit (Qiagen, Hilden, Germany) following manufacturer’s protocols. 2× 300 bp libraries for the Illumina MiSeq (Illumina, San Diego, CA) were prepared using the Illumina library preparation kit (see Supplemental Experimental Procedures, Supplementary Material online for detailed methods), and sequence data were checked for quality using FastQC (Andrews 2015). We supplemented our sequence data with previously published genomic data available for a *C. costatus* symbiont (Nygaard et al. 2016; Bioproject PRJNA295288 – 100610-02). We then assembled the genomes for each of the three data sets using four different software packages with default settings: 1) ABySS 2.0.0 (Jackman et al. 2017), 2) SGA 0.10.15 (Simpson and Durbin 2012), 3) SPAdes 3.7.1 (Nurk et al. 2013), and 4) SOAPdenovo 2.04 complemented with GapCloser 1.12 (Luo et al. 2012). All assemblies were further corrected for heterozygous regions and divergent diploid genomes using the package Redundans 0.12a (Pryszcz and Gabaldón 2016) (supplementary fig. S1, Supplementary Material online). This resulted in a total of eight assemblies for each data set. All assemblies were assessed for quality using several different genome statistics (table 1) which were generated using the ContigStats.pl script (written by Heath E. O’Brien, available at https://github.com/hobrien/Perl/blob/master/ContigStats.pl) and the software package BUSCO v4 (Simão et al. 2015).

**Table 1:** Genome Statistics for the Different Assemblies

Species/ID	Assembler ± Redundans	Total Length (bp)	No. of Contigs	N50 (bp)	Longest Contig (bp)	*N*’s	No. of Predicted Genes	Total BUSCO Genes (%)	BUSCO Genes Duplicated (%)
*Leucocoprinus* sp.	ABySS –	37,868,966	7,777	51,836	742,525	1,695,483	6,730	94.9	0.6
100610-02	ABySS +	41,010,461	2,091	111,840	1,288,898	96,942	7,054	97.5	0.7
	SGA –	94,552,020	48,075	3,297	92,288	0	14,239	83.6	0.4
	SGA +	64,486,262	5,151	31,855	524,303	95,670	9,385	96.5	1.5
	SOAPdenovo –	171,922,479	20,433	19,233	200,771	24,532,942	23,180	88.8	19.8
	SOAPdenovo +	190,471,773	10,447	30,961	285,616	2,602,102	26,171	96.0	26.1
	SPAdes –	101,100,038	27,307	9,377	213,758	533,869	14,882	91.4	0.4
	SPAdes +	79,165,892	4,326	75,775	496,909	104,891	11,069	97.5	1.0
*Leucocoprinus* sp.	ABySS –	37,642,602	9,676	16,119	234,386	223,574	6,794	94.4	0.5
MS140512-07	ABySS +	37,057,723	4,778	24,356	234,870	4,484	6,715	95.7	0.5
	SGA –	150,310,303	151,153	1,024	54,556	0	22,568	69.3	5.2
	SGA +	58,439,858	32,056	2,491	54,556	3,648	9,703	70.5	0.3
	SOAPdenovo –	80,942,739	30,870	4,169	42,629	450,122	11,837	67.3	0.3
	SOAPdenovo +	63,729,975	12,609	8,493	130,081	116,914	9,226	82.3	0.5
	SPAdes –	124,500,266	31,228	14,053	190,955	438,978	19,134	65.3	6.8
	SPAdes +	101,793,287	12,552	21,931	190,955	7,959	15,194	74.2	2.6
*Leucoagaricus barssii*	ABySS –	32,671,039	4,005	28,088	430,464	255,022	6,552	94.5	0.9
KM164561	ABySS +	33,544,974	2,248	42,155	558,264	4,497	6,641	95.7	0.5
	SGA –	78,616,683	74,089	1,143	54,770	0	15,165	66.3	4.9
	SGA +	43,854,981	25,027	2,392	54,770	3,356	9,083	67.8	0.5
	SOAPdenovo –	40,835,165	11,074	7,913	103,485	124,322	7,550	79.0	0.4
	SOAPdenovo +	39,685,961	4,810	19,634	245,171	40,103	7,252	89.6	0.5
	SPAdes –	58,776,147	24,243	4,952	120,587	398,844	11,519	64.7	3.8
	SPAdes +	47,589,435	8,392	10,680	120,587	11,950	9,023	81.1	1.4

Note.—Genome statistics as extracted using the ContigStats.pl script and the BUSCO pipeline. Assemblies with and without Redundans optimization are marked with – or +, respectively. Full BUSCO results are presented in supplementary table S1, Supplementary Material online.

Our analyses revealed considerable differences in the total length of the assemblies between the four assembler packages, with ABySS creating the smallest and most consistent assemblies (largest N50 and longest contig) for the *Leucocoprinus* sp. data sets, ranging in size from 37.1 to 41.0 Mb, both with and without Redundans. The other assemblers resulted in assemblies ranging from 41 Mb for *L. barssii* with SOAPdenovo to 172 Mb for the previously published *Leucocoprinus* sp. with SOAPdenovo. The largest differences in genome assembly lengths were found in the two ant fungal symbionts, indicating that the high heterozygosity levels found in these cultivars represent a challenge for most assemblers. Optimizing the data sets through Redundans reduced most of the assembly lengths and, with the exception of the SGA assemblies, also reduced the number of ambiguous nucleotides (*N*’s) in the assemblies.

**Fig. 1:**
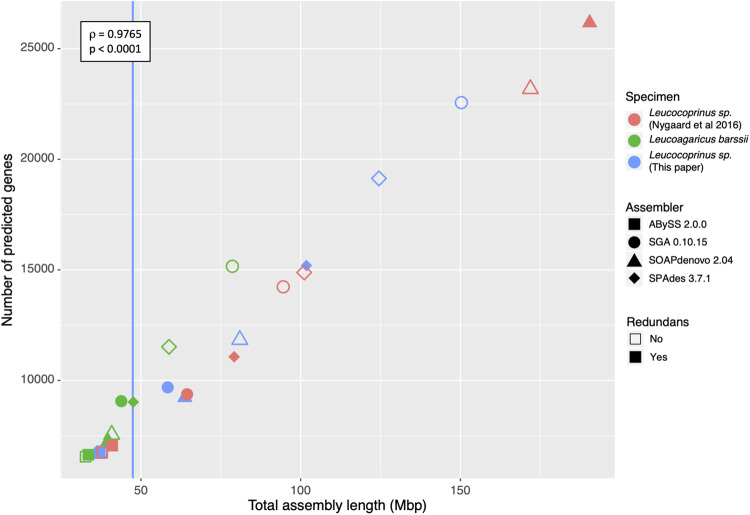
Correlation between genome assembly size and number of predicted genes. Based on the data obtained from the eight genome assemblies for each of the three samples, the number of predicted genes was positively correlated with the total assembly length (Spearman’s rank *ρ* = 0.9765, *P* < 0.0001). In most cases, optimizing the assembly using Redundans reduced the total genome assembly size presumably due to the removal of heterozygous regions. The one exception was observed for the previously published sequence data set from a *Cyphomyrmex costatus* fungal symbiont, *Leucocoprinus* sp., assembled with SOAPdenovo (Nygaard et al. 2016, in red). As indicated in the key, colors correspond to each of the three samples whereas the different shapes correspond to the four assemblers used, with filled shapes representing assemblies that also used Redundans, and open shapes corresponding to those which did not. The genome size for the fungal symbiont *Leucocoprinus* sp. isolated in this work and estimated using flow cytometry is marked by a blue line, showing that the best assemblies for this species were obtained using ABySS.

We then predicted genes for each of the assemblies based on genetic similarity using AUGUSTUS 3.2.2 (Stanke et al. 2004) trained with *Coprinopsis cinerea* (Schaeff.) Redhead et al. (2001), and extracted the number of genes with Genometools (Gremme et al. 2013, table 1). The total number of predicted genes correlated strongly with the length of the assembly (i.e., the longer the assembly the more genes were predicted; fig. 1; Spearman’s rank correlation *ρ* = 0.9765, *S* = 54, *P* < 0.0001). We note that our gene prediction numbers seem low compared with previously published genomes for other fungi belonging to Agaricales. One possible explanation could lie in the fact that the fungi grown by ants show similarities to endosymbionts, that is, the fungus is protected by the ants in underground chambers with barely any contact with the outside world. Endosymbiotic bacteria have been shown to have reduced genomes, both in size and number of genes (McCutcheon and Moran 2012) and a similar reduction could be happening in these fungi. A more plausible explanation, however, could be that the software used (AUGUSTUS) is not able to predict all genes using just DNA data, and that other techniques, such as the use of transcriptome data, or an increased sequence depth and genome coverage are necessary to recover all genes (Sims et al. 2014). Even so, these lower gene numbers do not detract from the overall observed pattern of higher numbers of genes with increased assembly length. As previously shown (Earl et al. 2011; Bradnam et al. 2013; Abbas et al. 2014), the assembly quality, based on N50, number of contigs and number of *N*’s, was also shown to depend on both the sample type (i.e., genetically heterozygous symbionts vs. free-living fungi) and, potentially, the genome size. It is therefore important to have prior knowledge of the genome size when embarking on any genome assembly project.

Because the different assemblers generated different assembly sizes, we estimated the genome sizes for the sequenced species using flow cytometry (Doležel et al. 2007). We isolated mycelium from three different ant colonies previously collected in Gamboa, Panama (*L. gongylophorus* from the ant *Atta colombica* Guérin-Méneville, 1844, *Leucocoprinus* sp. from *C. costatus*, and *Leucocoprinus* sp. from *Myrmicocrypta ednaella* Mann, 1922), and also from the oyster mushroom *Pleurotus ostreatus* (Jacq.) P. Kumm. (1871) collected at the Royal Botanic Gardens, Kew. We used *P. ostreatus* as the calibration standard for estimating the genome sizes in the other fungal species. However, because several genome size estimates are already available for *P. ostreatus*, rather than calculating an average value, we estimated its genome size directly, using *Arabidopsis thaliana* (L.) Heynh. (1842) (ecotype Columbia, Col-0, Galbraith et al. 1983; Bennett et al. 2003) as the internal standard (see Supplemental Experimental Procedures, Supplementary Material online for detailed methods). With a genome of 24.17 Mb (table 2), our estimate for *P. ostreatus* was shown to be similar to several previously published values (Kullman et al. 2005). All of the ant symbionts were found to have genome sizes which fell close to the global fungal average of 44.2 Mb/1C, with *L. gongylophorus* at 39.86 Mb, *Leucocoprinus* sp. from *C. costatus* at 47.17 Mb and *Leucocoprinus* sp. from *M. ednaella* at 49.10 Mb (table 2).

**Table 2:** Genome Size Estimation Using Flow Cytometry

Species	ID	1C-value (Mb)	Standard Deviation (Mb)	CV% (Standard)	CV% (Target)
*Pleurotus ostreatus*	KM237125	24.17	0.39	4.95	6.75
*Leucoagaricus gongylophorus* (*Atta colombica*)	Ac-2009-42	39.86	0.43	4.00	5.29
*Leucocoprinus* sp. (*Cyphomyrmex costatus*)	MS140512-07	47.17	0.10	3.94	4.45
*Leucocoprinus* sp. (*Myrmicocrypta ednaella*)	MS140507-01	49.10	0.79	4.24	5.23

Note.—Name of the ant species is given in parentheses below the fungal species name. The 1C-value represents the DNA content of the unreplicated haploid chromosome complement (i.e., the holoploid genome size sensu Greilhuber et al. 2005). CV% is the fluorescence peak width expressed as coefficient of variation.

Flow cytometry has been extensively used to estimate genome sizes over the last decades providing tens of thousands of estimates for eukaryotic organisms (e.g., Kullman et al. 2005 [fungi], Pellicer and Leitch 2020 [plants], Gregory 2020 [animals]). The advent and growth of sequencing technologies has meant that there is now an increasing amount of whole-genome short-read sequence data available, which are also increasingly being used to estimate genome sizes based either on k-mer analyses or by mapping short reads to contiguous assemblies (e.g., Sun et al. 2018; Pucker 2019). These novel estimates, however, sometimes differ with those obtained by flow cytometry, although the underlying causes are still somewhat unclear. The nature of the genome (i.e., presence of polyploidy, abundance and composition of repetitive DNA, and level of heterozygosity) could impose challenges when using standard bioinformatic pipelines. For this reason, large comparative analyses using both flow cytometry and genomic approaches on the same specimens are urgently needed (Pellicer and Leitch 2020) to understand what factors are responsible for the discrepancies observed and hence determine whether sequence data can ever reliably be used to provide robust genome size estimates.

**Fig. 2:**
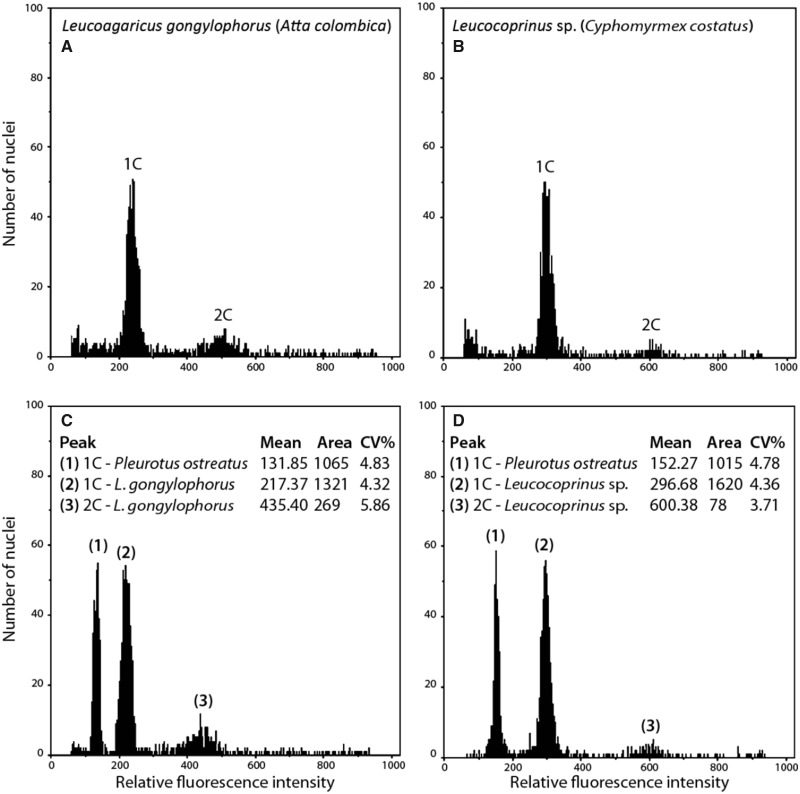
Examples of flow cytometry histograms used to estimate genome size. An example of the flow cytometry histograms obtained showing results for *Leucoagaricus gongylophorus* (*A* and *C*) and *Leucocoprinus* sp. (*B* and *D*) either without the standard *Pleurotus ostreatus* (*A* and *B*) or with (*C* and *D*).

Our flow cytometry histograms both showed high 1C (haploid) fluorescence peaks and low 2C (diploid) peaks (fig. 2). Because fungal nuclei are normally haploid and bearing in mind that we used fresh mycelium for analysis, the presence of 2C peaks indicates that some cells were in a premitotic division status (i.e., G_2_ phase of the cell cycle) at the time of measurement. An earlier study showing that the leaf-cutting ant fungus *L. gongylophorus* is functionally polyploid raised the possibility that each cell either contained multiple genomes within each nucleus (i.e., forming polyploid nuclei) or each individual nucleus contained just a single genome (i.e., forming haploid nuclei; Kooij et al. 2015). By extrapolating microsatellite data, it was suggested that each nucleus within this fungus was polyploid, however, our results suggest that the majority of nuclei analyzed are indeed haploid and hence the different genomes in each cell are present in different nuclei throughout the mycelium. Our results, therefore, suggest that the heterozygosity found in this fungus is most likely caused by SNPs in orthologs (i.e., genes from two different species with a common gene ancestor), possibly coming from divergent species, rather than paralogs (i.e., genes with different function arising from a gene or genome duplication event).

The genome size estimated by flow cytometry for the *Leucocoprinus* sp. from *C. costatus* is smaller than that estimated by most of the assemblies we obtained for this sample (table 1). In general, most assemblers using short-read sequence data have problems in compiling and assembling reads from long tandem repeat regions of the genome. Therefore, one might expect a smaller assembly size than the actual genome size, as has been reported in a previous study (Tavares et al. 2014). Based on the new genome size data generated using flow cytometry and various genome metrics such as total genome assembly length, N50, longest contig size and total percentage of BUSCO genes (see table 1), we conclude that out of the four assembly pipelines tested here, ABySS is the most accurate assembler for our samples.

In conclusion, our study has highlighted the importance of estimating genome size using flow cytometry prior to undertaking a whole genome sequencing and assembly project. This is essential given that prior knowledge of the genome size is essential to evaluate the quality of the genome assemblies and will avoid inferring incorrect gene number expansions, given the correlation observed between gene number and assembly size.

## Supplementary Material

Supplementary data are available at *Genome Biology and Evolution* online.

## Supplementary Material

evaa217_Supplementary_Data
