# Supplementary material for: Genome Size Versus Genome Assemblies: Are the Genomes Truly Expanded in Polyploid Fungal Symbionts?
Source: Genome Biol Evol. 2020 Oct 14;12(12):2384–90. doi: 10.1093/gbe/evaa217 (PMC7719231; doi:10.1093/gbe/evaa217)
Supplement: evaa217_Supplementary_Data [file evaa217_supplementary_data.zip › Kooij etal 2019 - SuppInfo-20200428.doc]

**Genome size vs. genome assemblies: are the genomes truly expanded in polyploid fungal symbionts?**

**Supplemental Information**

Pepijn W. Kooij^1,2*^ and Jaume Pellicer^1,3^

^1^ Department of Comparative Plant and Fungal Biology, Royal Botanic Gardens, Kew, Richmond, TW9 3DS, United Kingdom

^2^ Center for the Study of Social Insects, São Paulo State University (UNESP), Rio Claro-SP, 13506-900, Brazil.

^3^ Institut Botànic de Barcelona (IBB, CSIC-Ajuntament de Barcelona), Passeig del Migdia sn, Barcelona 08038, Spain.

^*^Corresponding and lead author: [p.kooij@kew.org](mailto:p.kooij@kew.org) at the above address

**Sample collections**

All ant fungal symbionts (*Leucocoprinus* sp Pat. (1888) MS140512-07 – host *Cyphomyrmex costatus* Mann 1922; *Leucocoprinus* sp MS140507-01 – host *Myrmicocrypta ednaella* Mann, 1922; *Leucoagaricus gongylophorus* (Möller) Singer (1986) Ac-2009-42 – host *Atta colombica* Guérin-Méneville, 1844) were isolated from colonies previously collected in Gamboa, Panama, and maintained at the Centre for Social Evolution at the University of Copenhagen. *Pleurotus ostreatus* (Jacq.) P. Kumm. (1871) (KM237125) was collected at the Royal Botanic Gardens, Kew, and deposited in the collection. Before drying fresh material was collected for culturing. Isolates were cultured on 39g/L Potato Dextrose Agar (Sigma-Aldrich, St Louis, MO, USA) with 5g/L Yeast Extract (Thermo Fisher Scientific Oxoid Ltd, Basingstoke, UK) until a full 5cm Petri dish was grown. A specimen of *Leucoagaricus barssii* (Zeller) Vellinga (2000) (KM162561) was sampled from the dried collections at the Royal Botanic Gardens, Kew.

**DNA extraction and sequencing**

Genomic DNA was extracted using the QIAGEN DNeasy Plant kit (Qiagen Ltd., Crawley, UK) following the manufacturer’s protocol. Samples were homogenised in lysis buffer for 30 seconds with a Zymo Research Terralyzer (Zymo Research, Irvine, CA, USA) in manufacturer provided ZR BashingBead Lysis Tubes (0.1 & 0.5 mm). Libraries for the two samples with an insert size of 550bp were prepared using the TruSeq Nano DNA LT (Illumina Inc., San Diego, CA, USA) sample kit following manufacturer’s instructions. We used a Quantus fluorometer with the Quantifluor ds-DNA system kit (Promega Corporation, Madison, WI, USA) to quantify the DNA in the extracts and libraries. We fragmented the DNA using an M220 Covaris ultrasonicator (Covaris Inc., Woburn, MA, USA) and evaluated the quality of the libraries with a 2100 Bioanalyzer (Agilent Technologies, Santa Clara, CA, USA). The libraries were sequenced (paired-end 2x300bp) with an Illumina MiSeq at the Royal Botanic Gardens, Kew. Resulting reads were quality checked using FastQC v0.11.3 (Andrews 2015), which gave an average PHRED score of 35 for both samples.

**Genome assembly and analyses**

Reads from the two new samples as well as publicly available data (Nygaard et al. 2016; Bioproject PRJNA295288 - 100610-02) for the *Leucocoprinus* sp symbiont of *C. costatus* were assembled without trimming using four different assemblers (Figure S1): (i) ABySS 2.0.0 (Jackman et al. 2017), (ii) SGA 0.10.15 (Simpson & Durbin 2012), (iii) SPAdes 3.7.1 (Nurk et al. 2013) and (iv) SOAPdenovo 2.04 complemented with GapCloser 1.12 (Luo et al. 2012). For ABySS, SGA and SOAPdenovo a standardised K-mer size of 31 bp was used, while SPAdes has a K-mer size optimiser built-in its software. To correct for heterozygous regions, all assemblies were further analysed with Redundans 0.12a (Pryszcz & Gabaldón 2016) using the default settings. From the resulting assemblies all sequences smaller than 500bp were removed as these tend not to be biologically meaningful. Genome assembly statistics were obtained using the ContigStats.pl script (written by Heath E. O’Brien, available at <https://github.com/hobrien/Perl/blob/master/ContigStats.pl>) and the software package BUSCO v4.0.0 (Simão et al. 2015). As a reference database for BUSCO we used the Agaricales_Odb10 database because each of the three samples fall within this order. We then predicted genes for each of the assemblies using AUGUSTUS 3.2.2 (Stanke et al. 2004) and extracted the number of predicted genes with GenomeTools 1.5.1 (Gremme et al. 2013). Scripts for all assemblies are available at <https://github.com/pwkooij/genome-scripts/blob/master/genome_analyses_scripts.sh>. The two new genomes sequenced in this study are deposited at NCBI under project number PRJNA560224. All genome assemblies are available at figshare (DOI: 10.6084/m9.figshare.8153204).

**Figure S1. Genome assembly pipeline used for this paper**

Each of the three sequence datasets were assembled using four different assemblers (ABySS, SGA, SPAdes and SOAPdenovo). SOAPdenovo assembly was always followed by GapCloser as described by the developers (Luo et al. 2012). Each of the assemblies was then optimised using Redundans. The resulting eight assemblies (the four original assemblies before Redundans and those after) were filtered to remove any sequence smaller than 500bp, as these tend not to be biologically meaningful. For each of the resulting eight filtered assemblies, genes were predicted using AUGUSTUS.

**Genome size estimation using flow cytometry**

Genome size was estimated using flow cytometry following the one-step protocol of Doležel, Greilhuber and Suda (2007) with minor modifications. We first estimated the genome size of *Pleurotus ostreatus* (Jacq.) P. Kumm. (1871) (KM237125) using *Arabidopsis thaliana* (L.) Heynh. (1842) ecotype Columbia (Col-0 1C = 156.48 Mb, Bennett et al. 2003) as internal standard. A small amount of mycelium was placed in a petri dish and co-chopped using a new razor blade with 0.5cm^2^ of *A. thaliana* in 2ml of the nuclei isolation buffer [LB01, Galbraith et al (1983)]. Samples were filtered through a 30μm nylon mesh, stained with propidium iodide (1 mg·ml-1; Sigma) to a final concentration of 50μg.ml-1, and kept on ice for 15min before measuring on a Partec Cyflow SL3 (Sysmex GmbH, Munster, Germany) flow cytometer fitted with a 100mW green solid state laser (Cobolt Samba, Solna, Sweden). In total, three samples were prepared, and each sample was run three times, recording the relative fluorescence of at least 1,000 nuclei per fluorescence peak (2C and 1C) for both the calibration standard and the target sample respectively. The resulting histograms were analysed using FlowMax software (v. 2.9; Partec GmbH). After confirming that the genome size of *P. ostreatus* (1C = 24.17 Mb) fell within the range of sizes obtained by Kullman (2000), we used this species as the internal standard to estimate the genome sizes of *Leucoagaricus* and *Leucocoprinus* following the same approach as described above.

**References**

Andrews S. 2015. FastQC - A high throughput sequence QC analysis tool. Available at: http://www.bioinformatics.babraham.ac.uk/projects/fastqc/.

Bennett MD, Leitch IJ, Price HJ, Johnston JS. 2003. Comparisons with *Caenorhabditis* (approximately 100 Mb) and *Drosophila* (approximately 175 Mb) using flow cytometry show genome size in *Arabidopsis* to be approximately 157 Mb and thus approximately 25% larger than the *Arabidopsis* genome initiative estimate of approximately 125 Mb. Annals of Botany. 91:547–557. doi: 10.1093/aob/mcg057.

Doležel J, Greilhuber J, Suda J. 2007. Estimation of nuclear DNA content in plants using flow cytometry. Nat Protoc. 2:2233–2244. doi: 10.1038/nprot.2007.310.

Galbraith DW et al. 1983. Rapid flow cytometric analysis of the cell cycle in intact plant tissues. Science. 220:1049–1051. doi: 10.1126/science.220.4601.1049.

Gremme G, Steinbiss S, Kurtz S. 2013. GenomeTools: A Comprehensive Software Library for Efficient Processing of Structured Genome Annotations. IEEE/ACM Trans Comput Biol Bioinform. 10:645–656. doi: 10.1109/TCBB.2013.68.

Jackman SD et al. 2017. ABySS 2.0: resource-efficient assembly of large genomes using a Bloom filter. Genome Res. 27:768–777. doi: 10.1101/gr.214346.116.

Kullman B. 2000. Application of flow cytometry for measurement of nuclear DNA content in fungi. Folia Cryptog Estonica. 36:31–46.

Luo R et al. 2012. SOAPdenovo2: an empirically improved memory-efficient short-read de novo assembler. Giga Sci. 1:18. doi: 10.1186/2047-217X-1-18.

Nurk S et al. 2013. Assembling single-cell genomes and mini-metagenomes from chimeric MDA products. J Comput Biol. 20:714–737. doi: 10.1089/cmb.2013.0084.

Nygaard S et al. 2016. Reciprocal genomic evolution in the ant-fungus agricultural symbiosis. Nat Commun. 7:12233. doi: 10.1038/ncomms12233.

Pryszcz LP, Gabaldón T. 2016. Redundans: an assembly pipeline for highly heterozygous genomes. Nucleic Acids Res. 44:e113. doi: 10.1093/nar/gkw294.

Simão FA, Waterhouse RM, Ioannidis P, Kriventseva EV, Zdobnov EM. 2015. BUSCO: assessing genome assembly and annotation completeness with single-copy orthologs. Bioinformatics. 31:3210–3212. doi: 10.1093/bioinformatics/btv351.

Simpson JT, Durbin R. 2012. Efficient de novo assembly of large genomes using compressed data structures. Genome Res. 22:549–556. doi: 10.1101/gr.126953.111.

Stanke M, Steinkamp R, Waack S, Morgenstern B. 2004. AUGUSTUS: a web server for gene finding in eukaryotes. Nucleic Acids Res. 32:W309–W312. doi: 10.1093/nar/gkh379.
